# Supplementary material for: Observations of the delayed-choice quantum eraser using coherent photons
Source: Sci Rep. 2023 Jun 16;13:9758. doi: 10.1038/s41598-023-36590-7 (PMC10275954; doi:10.1038/s41598-023-36590-7)
Supplement: Supplementary file 1 — Supplementary Information. [file 41598_2023_36590_MOESM1_ESM.pdf]

Supplemental Materials

Observations of the delayed-choice quantum eraser using coherent photons

S. Kim and B. S. Ham

Section A

Table S1. Raw data of the left column in Fig. 2.

No. data point	D1				D2				D1D2 coincidence			
	90	45	-45	0	90	45	-45	0	90	45	-45	0
181	25149	262	49274	25603	24593	49838	160	25132	89	2	2	92
182	25228	461	50002	25314	25003	49639	76	25336	91	4	1	92
183	24944	604	50294	25180	24771	49496	187	25187	89	5	2	91
184	25443	485	49485	25501	24732	49615	228	25426	90	4	2	93
185	24746	565	49283	25387	24922	49535	376	25357	89	4	3	92
186	24984	755	49255	25362	25113	49345	524	25297	90	6	4	92
187	25011	905	48813	25024	25146	49195	764	25146	90	7	6	90
188	24931	1172	48047	24954	24773	48928	772	25267	89	9	6	91
189	24840	1291	48073	25738	24874	48809	951	25367	89	10	7	94
190	25324	1596	48635	25508	24782	48504	1472	25501	90	12	11	93
191	24816	1584	48101	25131	25264	48516	1734	25273	90	11	12	91
192	25072	1783	47243	25326	25043	48317	1710	25306	90	13	12	92
193	24895	2459	47267	25362	24843	47641	2103	25590	89	17	15	93
194	24969	2583	47185	25554	25122	47517	2336	25603	90	18	16	94
195	25158	2993	47203	25273	24998	47107	2767	25069	90	21	19	91
196	24805	3374	46696	25381	24827	46726	3131	25394	88	23	21	93
197	24791	3762	46900	25292	24984	46338	3999	25183	89	25	27	91
198	24924	3888	46094	25149	25155	46212	4397	25559	90	26	29	92
199	25003	4339	45053	25171	25138	45761	4598	25035	90	29	30	91
200	25124	4821	44635	25131	25164	45279	5117	25568	91	32	33	92
201	24917	4904	43998	25517	24838	45196	5915	25015	89	32	38	92
202	25036	4863	43613	25498	24874	45237	6211	25092	89	32	39	92
203	25257	5600	43493	25445	25140	44500	6790	24973	91	36	43	91
204	24786	6647	43533	25139	24913	43453	7627	25778	89	42	48	93
205	25196	6904	42261	25401	25203	43196	8515	25263	91	43	52	92
206	25092	7530	42407	25292	25090	42570	8633	25437	90	46	53	92
207	25117	7940	41190	25248	25347	42160	9623	25364	91	48	57	92
208	25016	8498	40553	25335	25151	41602	10246	25035	90	51	60	91
209	25065	8781	40116	25272	25320	41319	10877	25284	91	52	63	92
210	24937	9805	40196	25772	25047	40295	11703	25306	90	57	68	94
211	24912	10758	39317	25460	25263	39342	12523	25146	90	61	71	92
212	24881	11342	38410	25656	24886	38758	13037	25416	89	63	72	94
⋮	⋮	⋮	⋮	⋮	⋮	⋮	⋮	⋮	⋮	⋮	⋮	⋮
360	25003	430	49840	25486	24998	49670	282	25564	90	4	3	94

Table S1 shows raw data in Fig. 2 for $0 \leq \varphi \leq 2\pi$. Each data includes five dark counts (per 0.1 s) in both D1 and D2. The data in Table S1 is from the recorded file by the homemade LV program. Figure S1 is for Poisson distributed single photons from an attenuated laser (SDL-532-500T, Shanghai Dream Laser; see Methods) by using neutral density (ND) filters. With appropriate NDs, a particular mean photon number can be set, where the measurement setup and measured photon streams are shown in Figs. S1(a) and (b). In the coincidence setup in Fig. S1(a), both single photon detectors (D1 and D2) count single photons independently, as shown in Table S2. The doubly bunched coherent photons are measured by the coincidence counting module (CCU; not shown) via D1 and D2, where the oscilloscope in Fig. S1 is replaced by a CCU. The box in Fig. S1(b) is to show the case of doubly bunched photons. By splitting each output path into two using an additional BS, four detector correlation scheme is configured for multiply bunched photon detection, whose data are shown in Fig. S1(c). The counted photon numbers depend on the laser intensity. From all measured counts, Fig. S1(c) shows single, doubly bunched, and triply bunched photons, satisfying Poisson distribution. For all individual data measured for one second, corresponding errors (standard deviation) are also calculated (‘sigma’ in Table S2). From Fig. S1(c), it is clear that a less mean photon number is better to avoid bunched photons. For example, the ratio of single to doubly bunched photons at 1 Mcps is 6440/1,000,000=6.4×10^{-3}. However, it increases to 750,000/10,000,000=0.075 for 10 Mcps. The ratio of triply- to doubly-bunched photon ratio is similar to the ratio of doubly-bunched to single photons at 25.6/6440=3.7×10^{-3}. Thus, the triply- or higher-order bunched photon cases are neglected compared to the doubly bunched one.

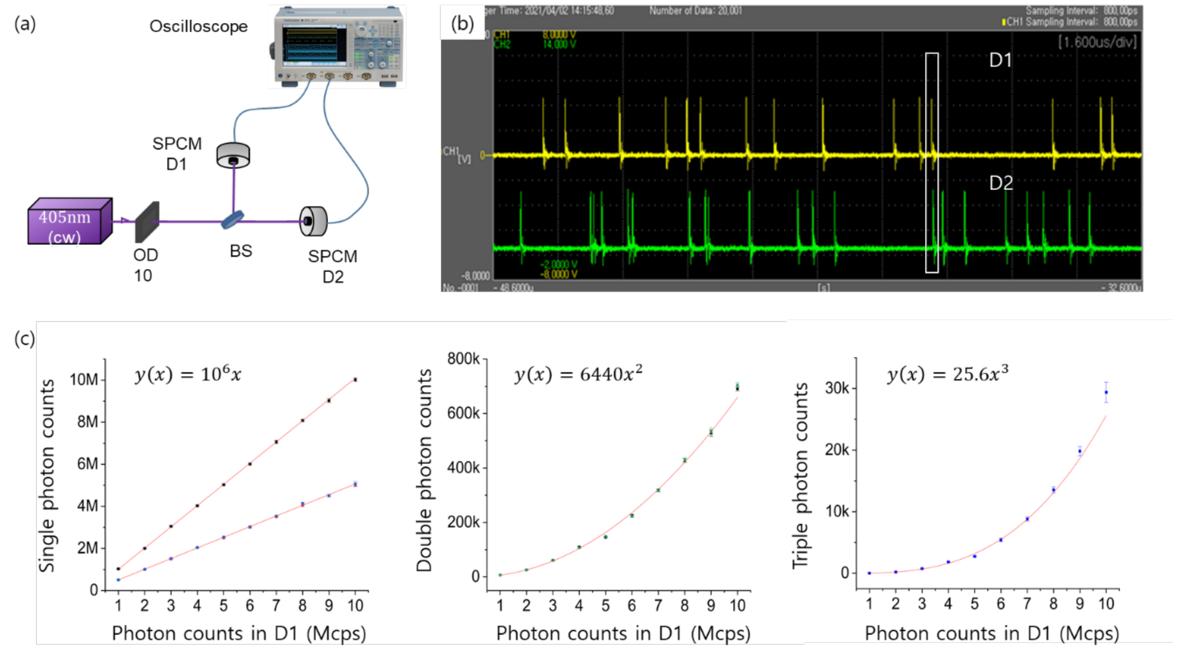

Figure S1. Poisson distributed photon characteristics. (a) Experimental setup for coincidence detection. (b) Single photon streams displaced on the oscilloscope. (c) Measured photon counts by CCU (see Table S2). The dots are data, and red lines are the best-fit curves.

Figure 2 of the main text is for a half million single photon case, resulting in a less than 1% bunched photon ratio. For the mean photon number calculation, the detected half million photons are divided by the dead time (22 ns)-caused time-slot numbers: $\langle n \rangle = \frac{0.5\times10^6}{4.5\times10^7} \sim 0.01$.

Table S2 shows the measurement results for UP and LP in Fig. 1 without BS and Ps to show coherent photon statistics. In each row, the average value (mu) and standard deviation (sigma) are for 30 individual samples of recorded data, where each sample is for 0.1 s accumulation time. Table S2 follows the Poisson statistics, as shown in Fig. S1(c), where the estimated standard deviation (see the shaded row) for D1 and D2 in Fig. 2 is less than 1 %. This extremely low error rate is the benefit of the coherent photons. The coincidence measurements (right two columns) are not for single photons but for doubly-bunched photon pairs, whose generation rate is <1 % of single photons.

Table S2. Photon statistics for Figs. 2 and 4.

D1		D2		D1D2 coincidence	
mu	sigma	mu	sigma	mu	sigma
counts/0.1s					
131.2	3.6	111.5	1.6	0.0	0.0
209.7	4.9	209.9	3.8	0.0	0.0
307.6	6.9	309.1	7.0	0.0	0.1
401.1	7.9	412.5	6.1	0.0	0.1
507.9	5.3	509.4	5.7	0.0	0.1
600.9	7.2	631.6	7.8	0.0	0.0
701.1	8.7	701.1	4.4	0.1	0.1
800.7	10.2	806	8.6	0.1	0.1
908.5	13.1	907.5	12.6	0.1	0.1
1008.8	8.8	1025.9	10.2	0.2	0.1
2429	15.0	2024.5	11.5	0.6	0.1
3015.9	17.0	3030	17.4	1.4	0.4
4014.5	20.8	4099.1	22.4	2.4	0.5
5010.3	40.6	5150.3	33.8	4.2	0.4
6085.6	35.4	6041.8	35.4	5.6	0.8
7010.2	41.7	7057.7	38.3	7.2	0.3
8055.6	45.9	8080.7	41.0	9.7	0.9
9102.8	53.8	9107.1	52.0	12.6	1.0
10100.1	62.2	10103.4	59.3	15.0	1.4
20040.1	87.4	20051.9	72.0	60.9	2.4
30200	100.8	30200.1	150.8	135.1	3.7
40600.1	160.5	40350.5	115.4	242.4	5.8
50500.1	223.1	50358.1	175.8	377.8	6.3
60699.5	313.6	61086.6	245.3	554.4	6.9
72000	382.0	71120.7	249.4	753.0	9.5
82000.1	420.2	82007.9	341.6	996.7	12.5
90036.6	452.4	90002.4	389.4	1197.2	18.6
100090.5	513.9	103092.7	482.1	1578.1	25.3

Section B

It is the well-known fact that the only mysterious quantum phenomenon is quantum superposition as Feynman mentioned [32]. Figure S2(a) shows a corresponding scheme to Fig. 1, where each MZI output in Fig. 1 is represented by superposition of orthogonal polarization bases with the same probability amplitudes. Figure S2(b) is an interferometric version of Fig. S2(a), where the second BS-caused phase shift ($\pi/2$) [33] can be controlled by φ adjustment. In Fig. S2(a), the split photon by BS satisfies quantum superposition between two paths of UP and LP, which are correlated with orthogonal polarization bases by the 90-degree rotated HWP in UP. The superposition of orthogonal polarization bases on the second BS in Fig. S2(b) is equal to that in Fig. S1(a).

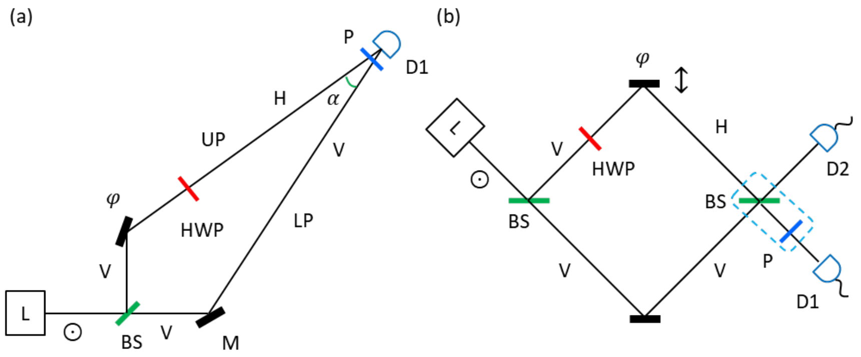

Figure S2. Equivalent schemes. (a) Classical beating-based. (b) MZI-based corresponding to (a). The dotted box has the same function as the EOM block in ref. [14].

In ref. [14], a linear optics-combined EOM block is used to control the MZI system for the delayed choice, e.g., to switch the particle nature with perfect which-way information to the wave nature with perfect visibility. Although the EOM block switching looks like a direct MZI control, it actually plays a role of P (45°) in Fig. 1 (see the dotted circle). Thus, the quantum eraser mechanism in ref. [14] is literally the same as Fig. 1. The causality violation is tested by post-measurements of the MZI output photons.

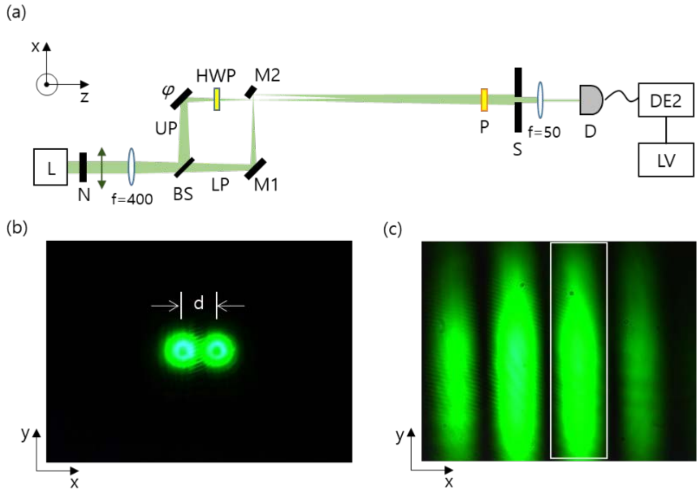

Figure S3. Schematic of Fig. S2(a). (a) Detailed configuration of Fig. S2(a). (b) Image of two beams (LP and UP) right after M2. (c) Image of interference fringe on the position S. L: laser, N: neutral density filter, BS: beam splitter, φ(PZT): piezo-electric transducer, HWP: half-wave plate, M1: mirror, M2: D-shaped mirror, P: polarizer, S: slit, D: single photon detector (or avalanche photon diode).

Figure S3(a) shows details of the experimental configuration of Fig. S2(a). The longitudinal distance between M2 and the slit S is 2 m. Figures S3(b) and (c) are the images captured by a CMOS camera placed M2 and S, respectively. In Fig. S3(b), the smallest separation (transverse distance, d) between two beams (UP, LP) is obtained by using a D-shaped mirror (M2), where the separation 'd' between UP and LP at M2 position is nearly diffraction limited at ~200 μm. Figure S3(c) shows the image of the spatial interference fringe on the position S caused by the d-separated two beams. For Fig. S4, only a center fringe (see the box in Fig. S3(c)) is taken through the slit S. The images of Figs. S3(b) and (c) are for cw laser L.

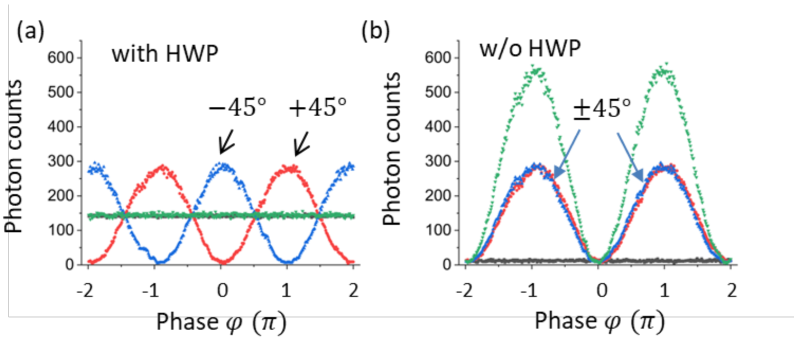

Figure S4. Experimental demonstrations for Fig. S3. (a) With HWP. (b) Without HWP. The single photon counts are for 0.1 s. Rotation angle θ of the polarizer P: Blue (−45°), Red (45°), Green (0°), Black 180°).

Figure S4(a) shows experimental results of Fig. S3(c) with single photons for four different rotation angles of P as a function of φ. For this, only center fringe of Fig. S3(c) is taken out through a homemade slit to the single photon detector D. Thus, the captured photon number is severely reduced compared with Fig. 2. As shown in Fig. S4(a), the polarizer's rotation angle θ gives the same effect as in the upper panels of Fig. 2, resulting in the same causality violation (see also Eqs. (6) and (7)). Like conventional delayed-choice experiments [13,14,19], the pre-determined photon characteristics have been retrospectively erased via post-measurements of the polarization basis control by P.

Figure S4(b) is for the same polarizers as in Fig. S4(a) without HWP, resulting in no causality violation. This is due to the preset wave nature of photons for the same polarization basis. Thus, Fig. S4(b) follows normal coherence optics of quantum superposition: $E_s = E_{UP} + E_{LP}$; $I_s = I_0(1 \pm cos\varphi)$. Here, the action of P in Fig. S4(b) simply reduces the observed photon counts into a half for $\theta = \pm 45°$. This fact is quite import to understand the measurement-caused quantum eraser in Fig. 2. For both interference fringes in Fig. S4, the MZI path-length difference in Fig. S3 is set to be far less than the coherence length of the laser.

For the particle nature, the bases must be distinguished as provided by PBS in Fig. 1 and HWP in Figs. S2 and S3. This feature seems to be the same as that of classical particles, but the paired bases via superposition between UP and LP require coherence between them, as shown in Fig. 2. For the wave nature, however, the bases must be indistinguishable, where the polarizer's rotation makes the polarization bases indistinguishable. Thus, the implied coherence of the particle nature can be recovered to the wave nature if the orthogonal polarization bases are controlled to be the same by the polarizer P. In other words, the initially preset orthogonal polarization bases of a single photon inside the MZI via quantum superposition are viewed or filtered out for the same polarization axis by the P's rotation (see Inset of Fig. 1). In this polarization projection process, 50 % photon loss is inevitable. Although the measurements are true for single photons, the controlled choices for the quantum eraser by Ps are for selected photons only. Such a 50 % loss is also inevitable in the time-bin entanglement measurements for coincidence detection [34,35]. For this, the MZI coherence must be kept to be $\Delta L \ll l_c$. Such phase coherence condition cannot be derived in conventional particle nature-based quantum mechanics. In that sense, classical particles with no coherence must be differentiated from quantum particles or single photons.

Section C

The PB-MZI in Figs. 1 and 2 are naturally stabilized under normal lab conditions. For this, the setup is just enclosed by a cotton box to minimize air fluctuations. As shown in Fig. S5, the PB-MZI is stabilized for as long as a few minutes, where the data collection time of each panel in Fig. 2 is 36 seconds.

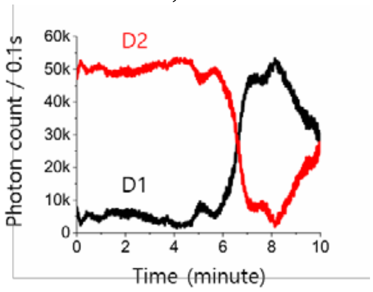

Fig. S5. PB-MZI stabilization.

Section D

Stokes parameter S of input light:

$$S = \begin{bmatrix} S_0 \\ S_1 \\ S_2 \\ S_3 \end{bmatrix}. \tag{S1}$$

Stokes parameter S of output light:

$$S' = \begin{bmatrix} S_0' \\ S_1' \\ S_2' \\ S_3' \end{bmatrix}. \tag{S2}$$

Mueller matrix M of a θ-rotated half-wave plate:

$$M = \begin{bmatrix} 1 & 0 & 0 & 0 \\ 0 & cos4\theta & sin4\theta & 0 \\ 0 & sin4\theta & -cos4\theta & 0 \\ 0 & 0 & 0 & -1 \end{bmatrix}. \tag{S3}$$

The horizontally polarized input light is represented as:

$$S = \begin{bmatrix} 1 \\ 1 \\ 0 \\ 0 \end{bmatrix}. \tag{S4}$$

Thus, the output Stokes vector of S through a θ-rotated half-wave plate is as follows (see https://www.youtube.com/watch?v=KHiOoThT1y8):

$$S' = MS = \begin{bmatrix} 1 & 0 & 0 & 0 \\ 0 & cos4\theta & sin4\theta & 0 \\ 0 & sin4\theta & -cos4\theta & 0 \\ 0 & 0 & 0 & -1 \end{bmatrix} \begin{bmatrix} 1 \\ 1 \\ 0 \\ 0 \end{bmatrix} = \begin{bmatrix} 1 \\ cos4\theta \\ sin4\theta \\ 0 \end{bmatrix}. \tag{S5}$$

For a half-wave plate rotated by $\theta = 22.5$, $\cos 4\theta = 0$ and $\sin 4\theta = 1$. From Eq. (S6), thus, the output light is diagonally polarized having both horizontal and vertical components:

$$S' = \begin{bmatrix} 1 \\ 0 \\ 1 \\ 0 \end{bmatrix}. \tag{S6}$$

Reference

32. Feynman, R. P., Leighton, R., Sands, M. *The Feynman Lectures on Physics*, Vol. III (Addison Wesley, Reading, MA, 1965).
33. Degiorgio, V. Phase shift between the transmitted and the reflected optical fields of a semireflecting lossless mirror is π/2. *Am. J. Phys.* **48**, 81-82 (1980).
34. Mercikic, I., de Riedmatten, H., Tittel, W., Scarani, V., Zbinden, H., Gisin, N. Time-bin entangled qubits for quantum communication created by femtosecond pulses. Phys. Rev. A **66** 062308 (2002).
35. Kwiat, P. G., Steinberg, A. M., Chiao, R. Y. High-visibility interference in a Bell-inequality experiment for energy and time. Phys. Rev. A **47**, R2472–R2475 (1993).
